# Monitoring of adverse drug reactions associated with antihypertensive medicines at a university teaching hospital in New Delhi

**DOI:** 10.1186/2008-2231-20-34

**Published:** 2012-09-10

**Authors:** Fowad Khurshid, Mohammed Aqil, Mohammad Shamshir Alam, Prem Kapur, Krishna K Pillai

**Affiliations:** 1 Faculty of Pharmacy, Jamia Hamdard (Hamdard University), New Delhi, 110062, India; 2 Pharmacoeconomics and Outcomes Research Unit; Department of Clinical Pharmacy, College of Pharmacy, King Saud University, P.O Box: 2457, Riyadh, 11451, Saudi Arabia; 3 Majeedia Hospital, Jamia Hamdard (Hamdard University), New Delhi, 110062, India

**Keywords:** Antihypertensive medicines, Adverse drug reaction, Teaching hospital and Pharmacovigilance

## Abstract

**Aim:**

To monitor the adverse drug reactions (ADRs) caused by antihypertensive medicines prescribed in a university teaching hospital.

**Methods:**

The present work was an open, non-comparative, observational study conducted on hypertensive patients attending the Medicine OPD of Majeedia Hospital, Jamia Hamdard, New Delhi, India by conducting patient interviews and recording the data on ADR monitoring form as recommended by Central Drugs Standard Control Organization (CDSCO), Government of India.

**Results:**

A total of 21 adverse drug reactions were observed in 192 hypertensive patients. Incidence of adverse drug reactions was found to be higher in patients more than 40 years in age, and females experienced more ADRs (n = 14, 7.29%) than males, 7 (3.64%). Combination therapy was associated with more number of adverse drug reactions (66.7%) as against monotherapy (33.3%). Calcium channel blockers were found to be the most frequently associated drugs with adverse drug reactions (n = 7), followed by diuretics (n = 5), and β-blockers (n = 4). Among individual drugs, amlodipine was found to be the commonest drug associated with adverse drug reactions (n = 7), followed by torasemide (n = 3). Adverse drug reactions associated with central nervous system were found to be the most frequent (42.8%) followed by musculo-skeletal complaints (23.8%) and gastro-intestinal disorders (14.3%).

**Conclusions:**

The present pharmacovigilance study represents the adverse drug reaction profile of the antihypertensive medicines prescribed in our university teaching hospital. The above findings would be useful for physicians in rational prescribing. Calcium channel blockers were found to be the most frequently associated drugs with adverse drug reactions.

## Introduction

Adverse drug reactions have been creating headlines over the last forty years since the thalidomide tragedy. International attention to patient safety has been growing significantly since the publication of the US Institute of Medicine report “To err is human: building a safer health system” [1-3]). There is no standard definition of an adverse drug reaction (ADR). Early studies used their own definitions, which were indistinct and could be interpreted to include intentional and unintentional overdose, as well as some administration errors [4,5]. According to World Health Organization (WHO) “An adverse drug reaction (ADR) is any response to a drug which is noxious and unintended and occurs at doses normally used in man for prophylaxis, diagnosis or therapy of disease or the modification of physiological function” [6]. Food and Drug Administration (FDA) defines a serious adverse event as one in which the patient outcome is death, or life threatening, hospitalization, disability, congenital anomaly or required intervention to prevent permanent impairment or damage [7]. ADRs are a major universal problem and are one of the leading causes of mortality and morbidity in health care facilities globally. The incidence of ADR varies with studies. A published meta-analysis of the incidence of adverse drug reactions (ADRs) in hospitalized patients concluded that ADRs rank as the fourth to sixth leading cause of death in the United States and the overall incidence of serious ADR accounted for 6.7% of hospitalized patients [8]. According to a study carried out at a private tertiary care hospital in South India, the incidence of ADRs was found to be 1.8%, out of which 12% of suspected ADRs were severe and 49% ADRs were moderate in severity [9]. A study by Arulmani et al. in India carried out in a secondary care hospital reported an overall 9.8% incidence of ADRs, of which 3.4% of ADRs were associated with hospital admissions [10]. Another study carried out in a tertiary care referral center in South India showed that admissions due to ADRs accounted for 0.7% of total admissions and deaths due to ADRs accounted for 1.8% of total ADRs [11]. Monitoring of ADRs is an ongoing, ceaseless, and continuing process. Though ADR monitoring is still in its infancy in India, this is likely to expand in the times to come. As the newer drugs are striking the Indian market, the need for ADR monitoring is growing more than ever before. Therefore, monitoring of the adverse effects particularly those of serious nature is obligatory [12]. It is important to remember that most ADRs would subside once the offending agent is discontinued or dosage reduced; however, many result in permanent damage. Therefore, it is important to motivate healthcare providers to understand their role and responsibility in the detection, management, documentation, and reporting of ADRs, and all essential activities for optimizing patient safety. The objective of this study was to monitor the ADRs caused by antihypertensive medicines prescribed in our university teaching hospitals.

## Methods

The present work was an open, non-comparative, observational study to reports incidence of ADRs due to antihypertensive medicines at our university teaching hospital. The study protocol was assessed and approved by Jamia Hamdard Institutional Review Board (Approval letter No. JHIRB 07/07, February 15, 2007). The study was conducted in patients attending the Medicine outpatient department (OPD) of Majeedia Hospital, a 150 bedded teaching hospital of Jamia Hamdard, New Delhi, India by conducting patient interviews after their informed consent was obtained and recording the data on ADR monitoring form as recommended by Central Drugs Standard Control Organization (CDSCO), Government of India (http://cdsco.nic.in/adr3.pdf). The information collected includes patient information (initials, age, sex, height, weight), suspected adverse event (brief description of the reaction, onset date/stop date of occurrence of events, outcomes of events , treatment receive), suspected medication (name, indication, start date/stop date, dose, frequency, route of administration), Medical history (past/present), concomitant medication, relevant test /laboratory data, other relevant history including pre-existing medical conditions. All hypertensive patients irrespective of age and sex and patients treated with at least one antihypertensive agent were included in the study. Patients who were not treated with antihypertensive agents, all the mentally retarded and unconscious patients (patients depending on other people for medication administration) and drug addicts were excluded from the study. All the data were kept confidential. The study was carried out during the period of February 2007 to May 2007 (4 months) by a registered pharmacist attending the medicine OPD on a daily basis. Study was conducted on 192 eligible patients at Majeedia Hospital who were willing to participate. Furthermore, some patients (n = 13) presenting with ADRs were observed for changes in biochemical parameters based on pathological lab reports.

The estimation of the probability that a drug caused an adverse clinical event is usually based on clinical judgment. For this study, the Naranjo’s scale which categorizes the causality relationship into *definite, probablepossible* or *unlikely* was used for the assessment of the exact nature of ADR [13].

## Results

During the study period, a total of 192 hypertensive patients visited Majeedia Hospital. Among the 192-hypertensive patients 87 (45.4%) were males and 105 (54.6%) were females. A total of 21 ADRs were observed in 13 out of 192 hypertensive patients. Among the 13 patients reported with ADRs 8 (4.1%) patients were female and 5 (2.6%) were male (Table 1).

**Table 1:** Frequency of different age groups in ADR and non ADR hypertensive patients

**Age groups (years)**	**Patient with ADRs**		**Patients without ADRs**		**Total**
	**Male**	**Female**	**Male**	**Female**	
21–30	0	0	12	3	15
31–40	1	0	19	28	48
41–50	1	5	10	33	49
51–60	0	2	23	13	38
61–70	1	0	11	13	25
71–80	2	1	5	7	15
81–90	0	0	2	0	2
**Total**	**5**	**8**	**82**	**97**	**192**

Females experienced more ADRs (n = 14, 7.29%) than males, (n = 7, 3.64%). The most vulnerable age group was 41–50 years with respect to ADRs (n = 6) followed by 71–80 years (n = 3), 51–60 years (n = 2), 31–40 years and 61–70 years (n = 1 each). No ADR was observed in the age group of 21–30 years and 81–90 years.

Out of 192 patients, 87 (45.3%) were receiving monotherapy and 105 (54.7%) were receiving multiple drug therapy. A significant difference in number of ADRs was observed in patients receiving monotherapy (33.3%) than those on combination therapy (66.7%). Calcium channel blockers (CCBs) was found to be the commonest therapeutic class associated with ADRs (n = 7), followed by diuretics (n = 5), β-blockers (n = 4), ARBs (n = 3) and ACE inhibitors (n = 2). Among individual drugs amlodipine was found to be the commonest drug associated with ADRs (n = 7) with one third of total number of reported ADRs. The common complaints with the usage of amlodipine were: abdominal pain, ankle oedema, sedation, pedal oedema, and back pain. Torasemide (a diuretic) was the next drug on the list of suspect drugs with 3 (14%) of total number of ADRs with fatigue, visual impairment and dizziness being the adverse effects. Dry cough was the most frequent ADR observed in our study with ramipril (Table 2).

**Table 2:** Adverse Drug Reactions and the suspected antihypertensive medicine

**Suspected Drugs (No. of Prescriptions)**	**ADRs Experienced**	No. of ADRs (%*)	**Interventions**
Calcium Channel Blockers			
Amlodipine (68)	Ankle edema	02 (2.94%)	Dechallenge
	Abdominal pain	02 (2.94%)	Symptomatic treatment
	Sedation	01 (1.47%)	Symptomatic treatment
	Pedal edema	01 (1.47%)	Symptomatic treatment
	Back pain	01 (1.47%)	Symptomatic treatment
Total		07 (10.29%)	
Diuretics			
Torasemide (18)	Fatigue	01 (5.55%)	Symptomatic treatment
	Visual impairment	01 (5.55%)	Dechallenge
	Dizziness	01 (5.55%)	No change in treatment
Total		03 (16.66%)	
Amiloride (09)	Dizziness	01 (11.11%)	No change in treatment
	Loss of appetite	01 (11.11%)	Symptomatic treatment
Total		02 (22.22%)	
Grand Total		05 (18.51%)	
ACE Inhibitors			
Ramipril (33)	Dry cough	02 (6.06%)	Dechallenge
Total		02 (6.06%)	
Angiotensine Receptor Blockers			
Telmisartan (17)	Dizziness	01 (5.88%)	No change in treatment
Losartan (22)	Dizziness	01 (4.54%)	No change in treatment
Olmesartan (06)	Dizziness	01 (16.66%)	No change in treatment
Total		03 (6.66%)	
Beta-blockers			
Atenolol (34)	Bradycardia	01 (2.94%)	No change in treatment
Metoprolol (12)	Headache	01 (8.33%)	No change in treatment
Propranolol (20)	Insomnia	01 (5.00%)	Dechallenge
	Depression	01 (5.00%)	Dechallenge
Total		04 (6.06%)	

*The percentage of ADRs was calculated from no of prescriptions of suspected drug.

On Naranjo’s probability scale more than half (57%) of the reported ADRs were classified as “possible”, 38% as “probable” and ~ 5% as “unlikely”. ADRs associated with CNS (n = 9, 42.8%) were found to be most frequent (e.g., dizziness, headache, depression etc.) followed by Musculo-skeletal (n = 5, 23.8%) complaints (e.g., back pain, fatigue ankle and pedal edema) and gastrointestinal (n = 3, 14.3%) disorders (e.g., abdominal pain, anorexia), (Table 3). Majority of ADRs observed in our study were mild (n = 14, 66.6%), which were well tolerated by the patients for example, headache, dizziness etc. followed by moderate (n = 7, 33.3%) ADRs, e.g. insomnia, depression (propranolol), ankle oedema (amlodipine) etc. The offending drug was withdrawn (dechallenged) which reversed the symptoms. None of the ADRs was categorized as severe.

**Table 3:** Organ system affected due to ADRs

**Organ System**	**No. of ADRs**	%* of ADRs
Central Nervous System	09	42.8
Musculo-skeletal System	05	23.8
Gastro-intestinal System	03	14.3
Respiratory System	02	9.5
Eye	01	4.8
Cardiovascular System	01	4.8
**Total**	**21**	**100**

*The percentage of ADRs was calculated from total no. of observed ADRs.

The biochemical parameters of the patients who experienced ADRs were mostly unperturbed. Only one patient who presented with ramipril associated dry cough had elevated Serum glutamate pyruvate transaminase (SGPT) and Serum glutamate oxaloacetate transaminase (SGOT) levels. The blood sugar levels were found to be more than the normal values only in patients with concomitant diabetes mellitus (Table 4).

**Table 4:** Biochemical parameters of the hypertensive patients exposed to ADRs

**S. No**	**Patient’s code**	**Age (Years)**	**Sex**	**Biochemical Parameters**							
				**SGPT 5–40 IU/L**	**SGOT 5–40 IU/L**	**B.Sugar 80–150 mg/dl**	**B.Urea 15–45 mg/dl**	**S.Creatinine 0.5–1.4 mg/dl**	**S.Sodium 136–149 meq/L**	**S.Potassium 3.5–5.4 meq/L**	**Chloride 98–108 meq/L**
1	HTN 01	38	M	15	12	121	21	0.8	144	4.5	105
2	HTN 02	50	F	37	27	189	32	1.3	144	4.2	106
3	HTN 03	46	M	27	33	159	74	1.8	135	5.1	106
4	HTN 04	73	M	27	33	290	16	0.9	138	4.8	98
5	HTN 05	47	F	16	12	343	15	0.8	139	3.9	102
6	HTN 06	58	F	40	42	122	32	1.0	141	4.4	103
7	HTN 07	45	F	37	28	180	32	1.3	144	4.2	104
8	HTN 08	45	F	26	29	250	38	1.2	140	4.3	100
9	HTN 09	75	M	34	42	148	18	0.9	141	4.5	105
10	HTN 10	80	F	27	29	130	25	1.2	140	4.6	102
11	HTN 11	63	M	27	38	189	30	1.3	140	4.8	104
12	HTN 12	60	F	30	28	110	26	0.9	139	4.3	103
13	HTN 13	50	F	68	81	107	17	1.0	146	4.6	108

**B. Sugar**: Blood Sugar, **B. Urea**: Blood Urea, **S. Creatinine**: Serum Creatinine, **S. Sodium**: Serum Sodium, **S. Potassium**: Serum Potassium, **SGOT**: Serum glutamate oxaloacetate transaminase, **SGPT**: Serum glutamate pyruvate transaminase.

## Discussion

The demographic details of our study population showed female gender predominance over males, which was similar to that reported in other studies found in the literature [10,11,14-18]. This might be due to higher emotion quotient in females, which makes them more sensitive to the pharmacological actions of medicines, thus enhancing the probability of ADRs. Rational dose titration may lead to minimization of ADRs in females. Incidence of ADRs was found to be higher in older patients i.e., more than 40 years (n = 12) as compared to younger ones i.e., less than 40 years (n = 1). Compromised organ functions, decreased BMR (basal metabolic rate), concomitant disease conditions and multiple drug regimens might be assigned as likely reasons for higher incidence of ADRs in older patients.

As anticipated, multiple therapies (more than one drug) were associated with more number of ADRs (67%) as against monotherapy (33%). Many epidemiological studies on risk factors for ADRs have shown that patients on multiple therapies were more likely to develop ADR as compared to patients on monotherapy [15,19-21]. Multiple therapies need to be discouraged as these enhance the probability of ADRs due to drug–drug interactions**.** It is recommended that only the absolutely essential medicines be prescribed in the management of hypertension.

CCBs were the most frequently associated drugs with ADRs. This is consistent with the findings of previous studies [16,22,23]. By contrast β-blockers have also been reported more significantly associated with ADRs than other drug categories [15,24] and the physicians considered discontinuing the treatment more frequently in patients receiving β-blockers in comparison to other drugs [15]. Among individual drugs amlodipine was found to be the commonest drug associated with ADRs. The common complaints with the usage of amlodipine were: abdominal pain, ankle oedema, sedation, pedal oedema, and back pain. Oedema has been reported elsewhere as the most common problem with amlodipine [16] and in other study conducted on 57 patients in Belgium [25]. Also, flushing, dizziness and peripheral oedema have been mentioned as common complaints with CCBs in a review [26]. Torasemide (a diuretic) was associated with fatigue, visual impairment and dizziness being the adverse effects. Dizziness and headache have been reported as common side effects associated with diuretics. These side effects could be related to the fluid or electrolytes imbalance caused by these medicines [26]. Dry cough was the most often ADR observed in our study with ramipril. This is in confirmation of previous reports with almost 44% of patients experiencing dry cough on using ACE inhibitors [22,27]. The adverse effects observed with other medicines were consistent with their pharmacological profiles.

The effects of ADRs on different organs/systems of the body were assessed and classified on the basis of symptoms reported by the patients who experienced adverse drug reactions. The most common systems associated with ADRs in our study were the central nervous system (CNS) followed by musculo-skeletal complaints. This finding is consistent with previous studies which have reported CNS manifestation [10,18,23,28]. The gastrointestinal system has also been reported to be involved in the majority of ADRs [10,18,23,29]. In our study, this formed the third largest report on ADRs.

The biochemical parameters of the patients who experienced ADRs were mostly unperturbed. Only one patient who presented with ramipril associated dry cough had elevated SGPT and SGOT levels. As the enzymatic levels were altered in an odd patient, it is concluded that the elevated SGPT and SGOT levels might be due to concomitant disease condition(s) and could not be attributed to ramipril administration. One patient receiving torasemide presented with fatigue and visual impairment along with elevated blood urea and serum creatinine levels. Again as the above observation was in merely one patient, the elevated blood urea and serum creatinine levels could be assigned to other pathological conditions and might not have been induced by torasemide. The blood sugar levels were found to be more than the normal values only in patients with concomitant diabetes mellitus.

## Conclusion

The above study is a part of ongoing pharmacovigilance program conducted at our university teaching hospital. During this pharmacovigilance study, calcium channel blockers were found to be the most frequently associated drugs with ADRs followed by diuretics, β-blockers, ARBs and ACE inhibitors. As the present study is related to ADR profile of antihypertensive agents, it may be helpful in selection of appropriate medicines for hypertensive patients, enhancing patient adherence with the therapy by selecting medicines of lesser ADRs profile, reducing unnecessary economic burden to the patients due to unwanted effects of the therapy.

## Competing interests

The author(s) declare that they have no competing interests.

## Authors’ contributions

FK collected the data in the Medicine OPD of Majeedia Hospital, New Delhi. MA conceptualized and supervised the research work and is the corresponding author. MSA prepared the manuscript. PK was the hospital clinician attached with the present study. KKP critically reviewed the manuscript internally prior to submission. All authors read and approved the final manuscript.
